# Adipose Stromal/Stem Cell-Derived Extracellular Vesicles: Potential Next-Generation Anti-Obesity Agents

**DOI:** 10.3390/ijms23031543

**Published:** 2022-01-28

**Authors:** Mariachiara Zuccarini, Patricia Giuliani, Valentina Di Liberto, Monica Frinchi, Francesco Caciagli, Vanni Caruso, Renata Ciccarelli, Giuseppa Mudò, Patrizia Di Iorio

**Affiliations:** 1 Department of Medical, Oral and Biotechnological Sciences, University of Chieti-Pescara, Via dei Vestini 29, 66100 Chieti, Italy; mariachiara.zuccarini@unich.it (M.Z.); patricia.giuliani@unich.it (P.G.); patrizia.diiorio@unich.it (P.D.I.); 2 Center for Advanced Studies and Technologies (CAST), University of Chieti-Pescara, Via L. Polacchi, 66100 Chieti, Italy; f.caciagli@unich.it; 3 Department of Biomedicine, Neuroscience and Advanced Diagnostic, University of Palermo, 90128 Palermo, Italy; valentina.diliberto@unipa.it (V.D.L.); monica.frinchi@unipa.it (M.F.); giuseppa.mudo@unipa.it (G.M.); 4 School of Pharmacy and Pharmacology, University of Tasmania, Hobart 7001, Australia; vanni.caruso@utas.edu.au; 5 Stem TeCh Group, Center for Advanced Studies and Technologies (CAST), Via L. Polacchi, 66100 Chieti, Italy

**Keywords:** obesity, metabolic disease/syndrome, extracellular vesicles, adipose tissue, adipose stromal/stem cells (ASCs)

## Abstract

Over the last decade, several compounds have been identified for the treatment of obesity. However, due to the complexity of the disease, many pharmacological interventions have raised concerns about their efficacy and safety. Therefore, it is important to discover new factors involved in the induction/progression of obesity. Adipose stromal/stem cells (ASCs), which are mostly isolated from subcutaneous adipose tissue, are the primary cells contributing to the expansion of fat mass. Like other cells, ASCs release nanoparticles known as extracellular vesicles (EVs), which are being actively studied for their potential applications in a variety of diseases. Here, we focused on the importance of the contribution of ASC-derived EVs in the regulation of metabolic processes. In addition, we outlined the advantages/disadvantages of the use of EVs as potential next-generation anti-obesity agents.

## 1. Introduction

Over the past two decades, obesity rates have risen dramatically in the developed and developing world [1,2], and the consequences of obesity represent a major public health concern [3].

Obesity is a complex and multifactorial medical condition resulting from a chronic imbalance between energy intake and energy expenditure, and characterized by increased adipose tissue stores [4]. Genetic and epigenetic signatures as well as behavioral and social factors contribute to the obesity epidemic [4]. Moreover, comorbidities associated with obesity, including cardiovascular and chronic kidney diseases, diabetes and some cancers greatly increase mortality rates in obese patients as well as public healthcare costs [5,6,7].

Health benefits in obesity result from weight loss. Pharmacotherapy is suggested as an adjunct to a reduced-calorie diet and increased physical activity for long-term weight management [8]. However, insufficient efficacy and concerns about drug safety for the long-term use of weight-loss agents quite often coincide with the termination of drug treatment and weight regain for the patient [8].

A large number of scientific investigations have focused on the role of adipose tissue (AT) in the pathophysiology of obesity in an attempt to identify new druggable targets. Several studies have highlighted the implications of multipotent mesenchymal stromal cells (MSCs) in the proliferation of the adipose mass in obesity [9]. MSCs are known as adipose stromal/stem cells (ASCs) [10] and are recognized as the primary source contributing to fat mass enlargement (reviewed in [11]). The metabolic activity of these cells is linked to the release of factors which are transported by different types of extracellular vesicles (EVs), also known as “nanoparticles”. Alterations of the physiological function of EVs have been linked to several human diseases, including those related to metabolic dysfunctions as well as blood and liver disorders (reviewed in [12]). For these reasons, the contribution of ASC-derived EVs in obesity could become a major focus in future drug discovery programmes.

Therefore, here, we reviewed recent literature on the role of EVs in the metabolism of AT, highlighting their specific contribution in adipogenesis and metabolic homeostasis. Additionally, we revised how the properties of ASC-derived EVs could be usefully exploited for potential therapeutic applications in obesity and related comorbidities.

## 2. AT: A Complex Tissue Compartment

The AT, widespread throughout the body, is a tissue of mesenchymal origin and it is usually divided into brown AT (BAT) and white AT (WAT).

BAT is known to be involved in the regulation of body temperature. Rodents and humans possess two types of thermogenic adipocytes arising from distinct lineages: the classical brown adipocytes and the so-called beige or brite cells that are released by the WAT to increase the body’s thermogenic capacity [13]. However, additional studies have demonstrated that BAT interacts with some peripheral tissues, secreting molecules capable of controlling their functions as well as body energy homeostasis [14].

In the human, WAT mainly acts as energy reservoir, storing fatty acids (FAs), which are released during fasting periods to supply energy to the organism. WAT is composed of subcutaneous AT (SAT) and visceral AT (VAT). Although morphologically similar, SAT and VAT show distinct characteristics with regard to metabolic homeostasis and dysfunction. While VAT expansion is associated with an increased risk of metabolic and/or cardiovascular diseases, as well as mortality, the expansion of SAT improves insulin sensitivity and reduces metabolic complications [15].

Human WAT is a heterogeneous tissue composed of 50% lipid-filled cells called adipocytes, which are involved in metabolic functions including lipogenesis, lipolysis and FA oxidation. However, these cells perform additional activities, behaving as immune and endocrine cells. In fact, they are able to release anti- and pro-inflammatory cytokines, as well as a large number of adipokines (reviewed in [15,16]), which modulate metabolism and the inflammation developed in obesity, thus playing a crucial role in the pathophysiology of this disorder. The remaining 50% is represented by non-adipocyte cells, which include endothelial and various types of immune cells, such as macrophages, eosinophils and mast cells. Collectively, this anatomical compartment of AT is referred to as the stromal vascular fraction (SVF), which is highly involved in the maintenance of AT homeostasis. A small but very relevant portion of this fraction is represented by ASCs. These cells possess multipotency and ability to give rise to osteoblastic, chondrocytic and adipocytic lineages [17]. Moreover, ASCs, both isolated from SVF and grown in culture, express mesenchymal surface markers in common with those of bone marrow-derived MSCs (BM-MSCs) such as CD90, CD73, CD105 and CD44, while they are negative for the hematopoietic marker CD45 (leukocyte common antigen) and CD31 (PECAM-1), a marker for endothelial cells also found in platelets and leukocytes. However, ASCs differ from BM-MSCs, being positive for CD36 (GPIIIb) and negative for CD106 (V-CAM-1) [17].

## 3. Features of ASCs in Healthy and Dysmetabolic Conditions

In an elegant study, Ong and colleagues identified several potential depot-specific markers by investigating over 240 cell-surface markers through high-content image screening. This study suggested that CD10 has a predominant expression in SAT-ASCs and CD200 in VAT-ASCs of both humans and mice. Additionally, the authors demonstrated that SAT-ASCs with high CD10 expression differentiated better towards an adipogenic phenotype than their low CD10 expression counterparts. In contrast, low-CD200 VAT-ASCs differentiated better than the same cells with high CD200 expression [18].

However, in the obese phenotype, there are further biological/functional differences regarding the ASCs [19]. In fact, the ASCs isolated from the SAT of obese subjects show a much higher proliferation rate and adipogenic differentiation capacity than those derived from VAT [20,21], while VAT-ASCs have upregulated clusters of genes related to lipid biosynthesis and metabolism [22]. Considering the limited ability of VAT-ASCs to differentiate into new adipocytes, this could partly explain the hypertrophy of existing adipocytes as a response to fat accumulation in obesity [23]. Conversely, the increased differentiation capacity of SAT-ASCs induces the formation of new adipocytes (hyperplasia) with smaller lipid vacuoles (reviewed in [24]), the number of which is approximately double in obese compared with non-obese subjects [25]. There are some exceptions, such as those shown by the ASCs present in mediastinal fat, which are considered to belong to the VAT. These cells express, in addition to putative mesenchymal surface markers, BAT-specific genes, with the capacity of differentiating into metabolically active brown adipocytes, which could contrast with fat accumulation in VAT [26].

**Figure 1:**
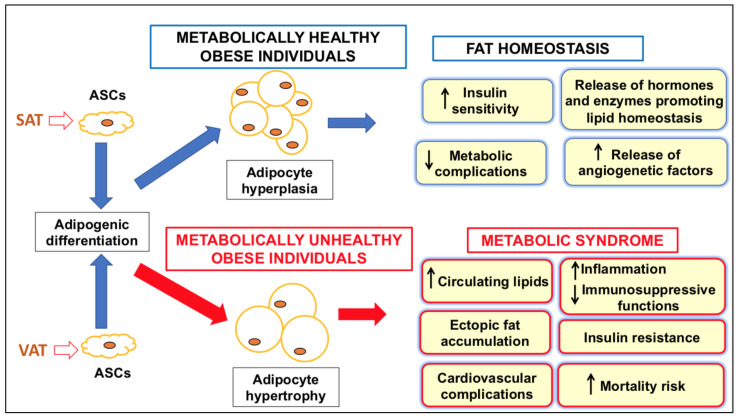
Schematic representation of the events occurring in metabolically healthy or unhealthy obese subjects. SAT-ASCs have the main function of regulating fat homeostasis in the AT, while VAT-ASC hypertrophy contributes to the onset of metabolic syndrome. Abbreviations: ASCs, adipose stromal/stem cells; SAT, subcutaneous adipose tissue; VAT, visceral adipose tissue.

SAT- or VAT-ASCs behave differently in obesity: a prevalent expansion of VAT, mostly due to ASC hypertrophy, is correlated with a greater susceptibility to metabolic disorders. However, this correlation is not observed in obese people with expanded SAT-ASCs, in whom the metabolic parameters are closer to the normal range, as reviewed in [11] and summarized in Figure 1. These subjects are indicated as “metabolically healthy obese”. However, most of them tend to lose their status over time, and obesity confers a higher risk of developing metabolic syndrome risk factors [27].

ASCs from the two adipose areas also differ in the expression/release of hormones linked to fat homeostasis, such as adipokines/lipokines, or the lipolysis/triglyceride synthesis rate [19,24]. Additionally, SAT-derived ASCs are mostly able to secrete angiogenic factors, mainly under hypoxic conditions, which allow cell survival in an ischemic environment. In vitro studies suggest that while these cells derived from healthy subjects act as a reservoir of growth factors necessary for promoting angiogenesis, this ability is impaired in ASCs derived from obese individuals [28].

ASCs usually exhibit immunosuppressive functions similar to most MSCs in healthy subjects; in contrast, in the AT of obese subjects, this function is compromised [29]. In agreement with these results, Lefevre et al. [30] showed that ASC depots exhibit specific differences in mice fed a high-fat diet so that low-grade inflammation develops with obesity in VAT, while it is delayed in SAT. In turn, the pro-inflammatory environment associated with metabolic disease influences ASC proliferation and differentiation potential. Thus, ASCs from obese animals, corresponding to an early stage of obesity, show an increase in senescence as well as in adipogenic potential compared with ASCs from lean animals. Conversely, the ASCs obtained from the SAT of morbidly obese patients, who represent a late stage in the obesity process, exhibited a lower capacity for proliferation and adipogenic differentiation than ASCs from non-obese subjects (reviewed in [28]). Altogether, these results highlight the heterogeneity of ASCs and indicate that the progression towards metabolic impairment can heavily affect all functions of ASCs [31,32]. These aspects are crucial factors for the use of ASCs in regenerative medicine, as pointed out in Section 5.

Recent investigations have suggested that ASCs from healthy or obese subjects can also be distinguished according to the biochemical proprieties of their EVs. In the next section, we report the most recent advancements in this regard.

## 4. Characterization of EVs Obtained from ASCs

EVs are nanoparticles that are gaining increasing interest as innovative tools in medicine, with a wide range of clinical applications in disease diagnosis, prognosis and therapy, as well as in tissue repair/regeneration [33]. They arise from the lipid bilayer of the membrane and are secreted from virtually all cells into biological fluids. EVs are heterogeneous in size, membrane composition and content, consisting of DNA, RNA, lipids and proteins. All these features largely represent the tissue or cell of origin [34]. Three main types of EVs have been characterized and labeled as exosomes, microvesicles and apoptotic bodies. The exosomes (sizes from 30 to 200 nm) are formed intracellularly and secreted after the fusion of multivesicular bodies with the cell surface; the microvesicles (200–1000 nm) are formed by the outward budding of the plasma membrane, while apoptotic bodies (larger than 1000 nm) are released during the late stage of apoptosis and mainly contain nuclear material from dying cells. There is also an additional subtype reported as shed midbody remnants, which are released during cytokinesis (200–600 nm) [35]. EVs have been identified as vital components in intercellular communication and being capable of transferring information to nearby or distant cells, which affects cells’ physiological and pathological functions [36,37].

In relation to AT, recent investigations have indicated that EVs released from WAT cells play a major role in maintaining metabolic homeostasis or modulating various metabolic disorders [12]. In addition, other studies suggested that BAT is also capable of releasing EVs, which could be used as therapeutic agents for obesity and related diseases [38]. Unfortunately, the availability and accessibility of these important AT depots are limited, so that the potential translational use of these ASCs and related EVs in clinical practice is reduced. Therefore, here, we focused on the content and activity of EVs, mainly exosomes, isolated from WAT-derived ASCs.

Standardized methodological approaches to isolate and characterize EVs from adipose cells as well as from any other sources are still lacking [39]. Taking this limit into account, a variety of nucleic acids and proteins have been identified in ASC-derived EVs as important mediators of the paracrine function of ASCs [40].

MicroRNAs (miRNAs) can be isolated from rat [41] and porcine ASC-derived EVs [42]. The miRNAs are small single-stranded non-coding RNA molecules (containing about 22 nucleotides) present in all living organisms that function in RNA silencing and post-transcriptional regulation of gene expression. In humans, the literature shows that the size, but not the number, of ASC-derived EVs from obese individuals is smaller than those from lean individuals [43]. Similar data have been obtained in swine [44]. Scientific evidence has indicated that obesity can alter the miRNA content of ASC-derived EVs as well as the ability of these nanoparticles to modulate important injury pathways in the recipient cells [43,45]. Data from human subjects showed that eight miRNAs were upregulated and 75 miRNAs were downregulated in obese EVs vs. lean EVs. While upregulated miRNAs from ASC-derived EVs of obese subjects were involved in the modulation of nuclear factor kB (NF-kB) and mitogen-activated protein kinase (MAPK) signals, as well as in cytoskeleton organization and apoptosis, those downregulated in the same EVs were implicated in the cell cycle and angiogenesis, and in modulating other signals such as those transduced by the Wnt and MAPK molecular pathways. The authors suggested that treating damaged human proximal tubular epithelial cells (HK2 cells) with ASC-derived EVs obtained from obese individuals were less effective at reducing inflammatory reaction and apoptosis than the corresponding EVs isolated from ASCs of lean subjects [43]. Similarly, intrarenal delivery of EVs obtained from ASCs of obese swine was unable to preserve the microcirculation and improve function in pigs with renovascular disease [45]. Collectively, these findings indicate that obesity might increase the content of these miRNAs in human ASC-derived EVs, which, in turn, might promote pro-inflammatory signals and apoptotic death, while reducing those involved in cell proliferation and angiogenetic processes.

Other studies have demonstrated that ASCs release exosomes that could influence proliferation and regeneration pathways in AT [46,47,48,49]. Among these articles, a recent review reported the presence of 591 proteins together with that of 604 miRNAs in EVs derived from human ASCs. The most important molecular function of these proteins is related to signaling receptor binding. The authors of this review also highlighted the biological processes in which these proteins are involved, which include development processes, cell adhesion, immune system processes, response to stimuli and locomotion, among others. As for the molecular functions of the miRNAs present in ASC-derived EVs, the authors substantially confirmed the involvement of these nanoparticles in the biological processes reported above [49].

## 5. Experimental Use of ASC-Derived EVs in Pathological Conditions

The content of ASC-derived EVs can be modified by various cell culture conditions, such as proinflammatory stimuli as well as oxidative stress or hypoxia, or by their transfection with lentiviral particles (reviewed in [49]), confirming their role in antigenic/anti-inflammatory/immunosuppressive functions [50]. Hence, EVs from ASCs are also currently actively studied for their possible therapeutic uses in several pathological conditions ranging from neurodegenerative to cardiovascular and respiratory diseases, as well as for regeneration of skeletal tissue or wound healing (Table 1) [47,49].

Special attention is paid to the angiogenic effects of ASC-derived EVs [51]. Pro-angiogenic effects could be ascribed to the presence of some miRNAs including miR-31, miR-125 and miR-126. Specifically, miR-31 suppresses the activation of the factor FIH-1 (factor-inhibiting hypoxia-inducible factor 1), thus favoring the activation of hypoxia-inducible factor 1 (HIF-1), a key regulator that mediates the cell response to hypoxia and angiogenesis. miR-125 inhibits the expression of the angiogenic inhibitor delta-like 4, and miR-126 maintains a proliferative reserve in endothelial cells through suppression of the Notch1 inhibitor delta-like 1 homolog (Dlk1) [52].

**Table 1:** Some of the experimental uses of EVs derived from ASCs for clinic applications.

Diseases	Model	Effects	Refs.
**NEURODEGENERATIVE**			
Alzheimer’s disease	CT	N/A (NCT04388982)	[47]
	Transgenic mouse	Increase in neuron survival.	[53]
	Cell lines	Reduction of ß-amyloid levels and deposition.	[54]
Amyotrophic Lateral Sclerosis	Neurons from WT or G93A ALS mice.	Restoration of mitochondrial proteins	[55]
Huntington’s disease	Neurons from transgenic (R6/2) mice	Reduction of aggregate accumulation and mitochondria dysfunction	[56]
Multiple Sclerosis	Mice with experimental encephalomyelitis	Remyelination promotion coupled to lymphocytes Th1 and Th17 reduction	[57]
Ischemic stroke	Rats with ischemic brain injury	Reduction of cerebral infarct volume and neuroprotection	[58]
**RESPIRATORY**			
Pulmonary emphysema	Mice with experimentally-induced emphysema	Inhibition of emphysema by an FGF2-dependent pathway	[59]
Acute lung injury	Histone-mediated lung injury in mice	Improvement of pulmonary inflammation via PI3K/Akt pathway	[60]
Pulmonary infection	CT	N/A (NCT04544215)	[47]
COVID-19 respiratory distress	CT	N/A (NCT042276987)	[47]
**VASCULAR**			
Experimental studies on vascularization	In vitro (cell cultures) and in vivo (mouse) models	Promotion of VEGF secretion from endothelial cells and of neo-angiogenesis;	[61,62]
		Increased content of various miRNAs promoting vascularization	[26,51,52,63]
**OSTEOMUSCULAR**			
Muscle acute ischemia	Murine model of hindlimb ischemia	PDGF-induced expression of anti-inflammatory factors protecting muscles from ischemia	[64]
Skeletal tissue regeneration	Myoblast cell line	Promotion of muscle cell functions due to miR-21	[65]
Osteoarthritis	Cell cultures and murine model of osteoarthritis	Induction of osteogenic differentiation and suppression of inflammation by up-regulation of miR-145 and -221	[66,67,68]
Torn rotator cuffs	Rats with rotator cuff tear	Prevention of muscle degradation	[69]
**SKIN**			
Skin flap recovery	Ischemic flaps in rats	Reduction of inflammation and cell apoptosis	[70]
Plastic surgery	Brown Norway-to-Lewis rat hindlimb transplantations	Increased tissue survival by CD4+ T and Th1 lymphocyte down-regulation coupled o Tr1 and Treg upregulation	[71]
Wound healing	Cell cultures and mouse model of skin trauma	Increase in cell migration and proliferation by miR-21, which enhanced MMP-9 expression via PI3K/Akt pathway, or by miR-19b, which promoted wound healing via TGF-ß signal	[72,73]
	Diabetic rats with full-thickness excision wound	Promotion of wound repair by engineered ASC-EVs containing miR-21-5p able to stimulate Wnt/ß-catenin pathway	[74]

Abbreviations: ALS, amyotrophic lateral sclerosis; ASCs, adipose stromal/stem cells; CT, clinical trial; EVs, extracellular vesicles; FGF2, fibroblast growth factor 2; N/A, not available; PDGF, platelet-derived growth factor; PI3K, phosphatidylinositol 3-kinase; TGF-ß, transforming growth factor ß; VEGF, vascular endothelial growth factor; WT, wild-type.

## 6. Experimental Use of ASC-Derived EVs in AT Homeostasis and Disease

Jung et al. isolated EVs from human ASCs during white or beige differentiation in vitro, observing that EVs from both cell sources could induce the successful differentiation of other ASCs into white or beige adipocytes, promoting adipogenesis in vivo as well [75]. Indeed, intraperitoneal injection of EVs from ASCs that differentiated into white adipocytes promoted the regeneration of AT in mice after 4 weeks, resulting in increased expression levels of specific adipogenic markers such as peroxisome proliferator-activated receptor γ (PPARγ), fatty acid binding protein 4 (FABP4) and leptin.

On the other hand, BAT-derived EVs prevented diet-induced obesity (DIO) in mice fed a high-fat diet by causing browning of AT while suppressing the accumulation of lipid droplets in liver tissue and restoring glucose homeostasis. The miRNAs contained in these nanoparticles were primarily responsible for the observed effects of BAT-derived EVs. Accordingly, mice with adipose-tissue-specific knockout of the miRNA-processing enzyme Dicer (ADicerKO), as well as humans with lipodystrophy, showed decreased levels of circulating exosomal miRNAs, while transplantation of WAT or BAT into ADicerKO mice restored the level of many circulating miRNAs. This event was associated with an improvement in glucose tolerance and a reduction in hepatic Fgf21 mRNA and circulating FGF21, a hormone that regulates important metabolic pathways [76].

Therefore, EVs secreted during stem cell differentiation into white or beige adipocytes can promote cell reprogramming, acting as a pivot point in fat homeostasis.

Zhao et al. [77] demonstrated that treating obese mice with ASC-derived exosomes isolated from the fat pad of normal-weight mice improved insulin sensitivity, while reducing obesity and hepatic steatosis in these animals. These findings were mainly based on the transfer of EV content to the macrophages, directing them towards an anti-inflammatory M2 phenotype. Interestingly, exosomes also contain high levels of tyrosine hydroxylase, which is responsible for catecholamine release and lactate production, both events favoring the transition of WAT to a beige phenotype and improving metabolic homeostasis in response to the challenge of high-fat food. These findings outline a novel exosome-mediated mechanism for cross-dialogue between ASCs and macrophages.

Notably, the anti-inflammatory behavior of ASC-derived EVs could also be exploited in inflammatory bowel disease (IBD), for which there is no effective and safe treatment. Yu and colleagues [78] recently reported in a mouse model of IBD that exosomes from human ASCs protected the integrity of the gut by activating the proliferation of intestinal epithelial and stem cells. Moreover, the anti-inflammatory/immunomodulatory potential of ASC-derived EVs is now being actively investigated for possible applications in acute and chronic inflammatory conditions, including experimental allergic airway models, ultraviolet B ray exposure-induced skin alteration or inflammation associated with traumatic brain injury [79,80,81].

Taken together, these data indicate that EVs could play an important role in immune and metabolic homeostasis in WAT, while also providing a novel tool to control inflammatory processes in obesity [82].

**Figure 2:**
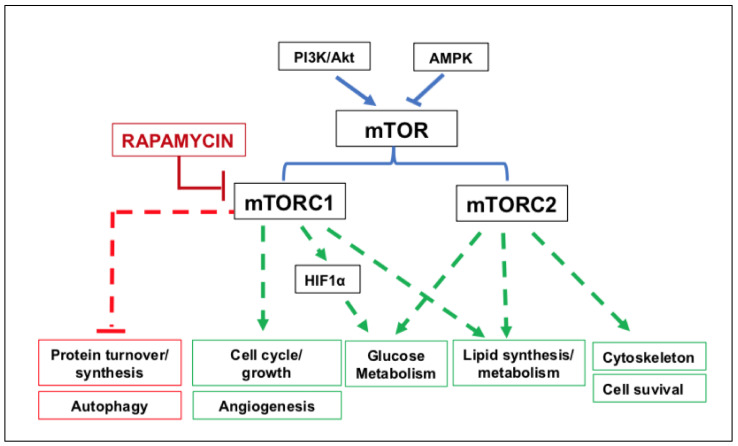
Scheme of the activities promoted by the mTOR (mammalian target of rapamycin) system, composed of two complexes known as mTORC1 and mTORC2, and of its modulation by the phosphatidylinositol 3-kinase (PI3K) and AMP-activated protein kinase (AMPK) pathways. Rapamycin exogenously inhibits the mTOR system, mostly acting on the activities of mTORC1. Arrows drawn with dashed lines indicate that more downstream factors contribute to the final effect caused by the activation of mTORC1 and mTORC2. Abbreviation: HIF1α, hypoxia-inducible factor 1α.

Additionally, the therapeutic potential of ASC-derived EVs has been investigated in models of obesity-related comorbidities such as diabetic nephropathy [83] or delayed wound healing [84]. The administration of ASC-derived exosomes attenuated the negative consequences of spontaneous diabetes in mice, reducing the levels of urinary proteins, serum creatinine, blood urea nitrogen and podocyte apoptosis, which improved the clinical picture of renal insufficiency. The authors ascribed this improvement to the presence of miR-486 within EVs, a key factor capable of reducing the activation of the mammalian target of rapamycin (mTOR). mTOR is a serine/threonine protein kinase belonging to the phosphatidylinositol 3-kinase (PI3K)-related protein kinases (PIKK) family. In mammals, it is the catalytic subunit of two protein complexes known as mTOR complex 1 (mTORC1) and 2 (mTORC2), which have different levels of sensitivity to rapamycin and whose activities are summarized in Figure 2.

Thus, in rats with diabetic nephropathy, inhibition of this molecular pathway by miR-486 led to an increase in autophagy, a protective process that is important for maintaining normal cell homeostasis, and a reduction in podocyte apoptosis.

ASC-EV effects, tested in both in vitro and in vivo models, contributed to diabetic wound healing by promoting angiogenesis via the HIF-1α signaling cascade. In this case, the positive effect was due to the activation of the mTOR complex, with the contribution of the PI3K/Akt pathway [84].

Of note, PI3K/Akt and mTOR signals are interconnected and activated by the presence of high levels of insulin and insulin-like growth factor 1 (IGF-1) in overweight/obese subjects [85]. Conversely, caloric restriction inhibits PI3K/Akt/mTOR pathway activation, in part at least, via AMP-activated protein kinase (AMPK) activation [86]. While the interaction between ASC-derived EVs and PI3K and/or AMPK signals needs to be explored, the observations reported above indicate that these nanoparticles, at least in cells that are different from adipocytes, can contribute to the homeostasis of their functions by involving the mTOR pathway. Thus, it would be of interest to explore whether ASC-derived EVs can influence mTOR signaling directly, acting at the adipocyte level, as mTOR is crucial in regulating energy balance/body weight [87] and fat/glucose homeostasis [88].

**Figure 3:**
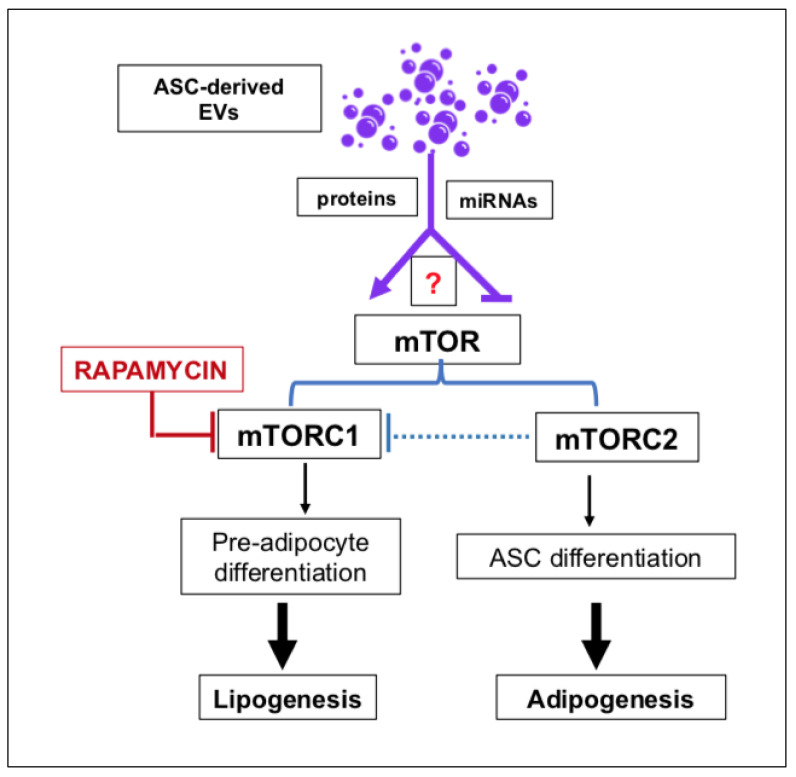
ASC-derived EVs could exert a modulatory role on the activity of the two components (mTORC1 and mTORC2) of the mTOR system in cells of the white adipose tissue (WAT) through the release and donation of selected molecules (miRNAs, proteins). The arrow drawn with a dotted line indicates that mTORC2 modulates mTORC1′s function, probably inhibiting it. The red question mark indicates that EV-mediated activities have yet to be defined in adipocytes. Abbreviations: ASCs, adipose stromal/stem cells; EVs, extracellular vesicles; miRNAs, microRNAs.

Specifically, mTORC1 has long been recognized as an epigenetic and post-translational modulator of lipid turnover, and its stimulation promotes the differentiation of pre-adipocytes in mature cells by activating PPARγ. This process enhances lipogenesis, that is, fat accumulation into lipid vacuoles. More recently, mTORC2 activity has been shown to promote adipogenesis, that is, the differentiation of ASCs into pre-adipocytes. mTORC2 also plays a modulatory role on the activity of mTORC1 (reviewed in [87]). Therefore, global deregulation of the mTOR system remarkably alters, among others, lipid homeostasis, leading to the development of obesity. Accordingly, the use of the classic mTOR inhibitor, rapamycin, in cultured human pre-adipocytes inhibited their complete differentiation into adipocytes [89] (see also Figure 3).

However, this effect was time-dependent. Indeed, only long-term blockade of mTORC1 by rapamycin can reduce DIO in mice [90]. In contrast, it has been reported that the pretreatment of human ASCs with the same drug enhanced autophagy, while reducing apoptosis, delaying senescence, promoting adipogenesis of those cells in vitro and increasing their survival and angiogenesis in vivo [91]. These effects could be explained by the greater sensitivity of mTORC1 to inhibition by rapamycin, which leads to the prevalent activity of mTORC2 (Figure 3). In this scenario, it would also be desirable to investigate which and how much of these effects are mediated by molecules donated from ASC-derived EVs to neighboring cells through the involvement of the mTOR system.

## 7. Advantages and Disadvantages of the Potential Use of ASC-Derived EVs in the Clinic

Research on EVs is producing substantial results in obesity, attracting pharmaceuticals to develop EV analogs. However, to our knowledge, there is no approved product for any therapeutic application at the present, while EVs, mainly exosomes, are being used for diagnostic purposes (reviewed in [92]). In particular, ASC-EVs could be exploited as a diagnostic tool for the assessment of obesity and the related metabolic complications. In fact, EVs from obese patients are characterized by the presence of specific biomarkers in their content, such as perilipin A, which is associated with insulin resistance, or cystatin C and CD14, which are associated with cardiovascular complications (reviewed in [93]).

Clearly, the therapeutic use of EVs from ASCs in the clinic is also attractive and scientifically supported by the results obtained through a wide variety of experimental pathological conditions, ranging from kidney transplantation to inflammatory bone diseases and tissue regeneration [94,95,96,97].

The use of EVs for clinical applications appears to offer several advantages over that with adult stem cells. However, the evidence is not univocal, and there are conflicting results, which exist even for the clinical use of MSCs (see Table 2).

The important features of EVs for their potential application in experimental therapy include good handling [98,99], which should not cause pulmonary embolism, as this can occur with ASCs, when administered in large numbers [100]. The EVs’ small size also allows them to cross the blood–brain barrier (BBB) easily after systemic administration, while their low/null expression of membrane histocompatibility markers reduces the risk of host immune responses [99]. Moreover, EVs are more resistant to injuries caused by freeze–thaw cycles and show high in vivo stability [99].

However, to date, the methods of EV isolation are not yet fully standardized and, therefore, there is a problem regarding their purification and identification, together with another problem related to the storage stability of EVs in view of their possible therapeutic application [108]. In addition, a general limitation for all potential applications of EVs in therapy is the age of the tissue/cell donors. As with AT, it has been reported that the number of ASCs obtainable from SAT decreases with age [101]; probably, the same happens for ASC-derived EVs [44]. Unfortunately, results on the differences in EV characteristics obtained from young and senescent cells are still conflicting [101]. Another important issue is the weight of the donors [102], as well as the presence of metabolic pathologies [29], which reasonably alter the quality and characteristics of the EVs obtained from those cells [109].

Furthermore, it must be considered that EVs carry only a fraction of the molecules produced by the cells of origin, which are selected for specific purposes such as supplying neighboring/distal cells with energetically, biochemically or enzymatically useful materials, as well as nucleic acids, which can alter the expression of the target genes. This can be an advantage but also as a disadvantage, considering the restriction in the range of products donated by EVs compared with entire MSCs [96], and also implies that the therapeutic dose and efficacy of EVs must still be clearly defined. Furthermore, EVs can home in on certain tissues depending on their source tissue, which would be mainly useful in the case of EV use for targeted drug delivery [33], although it is not clear how extensive this homing ability is.

Notably, MSCs can undergo spontaneous transformation and may therefore be tumorigenic. A decreased carcinogenic potential has been shown by induced pluripotent stem cells (iPSCs) [103], which are pluripotent stem cells generated from somatic cells. iPSCs can be differentiated into MSCs and expanded to generate a large population of homogeneous MSCs. However, further studies are warranted to determine the safety of iPSCs before their use in clinical trials. The same point should be considered for EVs, which do not have carcinogenic characteristics [98], although we should not overlook the fact that, similar to ASCs, EVs can also present a tumorigenic risk. For example, in patients with ovarian cancer, omental AT, which contains numerous ASCs, appears to promote tumor growth via paracrine secretion supported by ASC-derived EVs [106]. In fact, these EVs, secreted in the extracellular space, allow communication between tumor cells and ASCs with macrophages and immune cells, potentially favoring cancer progression and drug resistance. Again, as reported above, ASC-derived EVs show angiogenic properties [50], which may also favor tumor expansion and metastasis. However, there is also some opposing evidence. Thus, in immunocompetent mouse models of breast cancer, Li and colleagues [105] identified ASCs endowed with a typical mesenchymal stem marker, CD90, which had different expression levels, namely CD90^high^ and CD90^low^ ASCs. Interestingly, the CD90^low^ cell subset and its derived EVs significantly inhibited tumor growth in tumor-bearing mice, decreasing cancer cell proliferation and migration and enhancing their apoptosis. Therefore, EVs derived from ASCs and other stem cell sources could also serve as a new anticancer tool.

Finally, it should be noted that obesity severely modifies the properties of EVs, enhancing their inflammatory profile [49] as well as the activity of HIFs contained in ASC-derived EVs [51], which could also promote metabolic diseases, as observed in diet-induced disease models [107]. Thus, biotechnological modification of the EVs aimed at eliminating these factors could be useful for the treatment of those disorders. In particular, EVs can be engineered to generate EVs with modified/enhanced therapeutic properties or tissue tropism [110]. Additionally, EVs can be helped to find target cells/tissues by adopting delivery alternatives such as hydrogel encapsulation to favor locally sustained persistence of these nanoparticles [111].

## 8. Conclusions

In conclusion, ASC-derived EVs play an important role in the modulation of metabolic processes and could be exploited as a valid diagnostic tool in obesity and in metabolic syndrome, for the management of which they also represent an attractive cell-free therapeutic tool.

These clinical applications undoubtedly open up a fascinating scenario for the development of a new generation of anti-obesity agents. However, although the safe/appropriate use of EVs in clinical practice appears feasible and tangible, standardized good manufacturing practices (GMPs) for the clinical GMP-grade production of EVs are needed for the optimization of cell cultures, purification, quantification and quality control [112,113].

## Figures and Tables

**Table 2:** Advantages and disadvantages of the use of EVs for future therapeutic/clinical applications.

Advantages	Refs.	Disadvantages	Refs.
EV use for diagnostic purposes	[92,93]	Methods of EV isolation are not yet fully standardized	[98]
Good handling	[98]	EVs’ storage stability is not well known	[98]
Small size precluding pulmonary embolism if administered in a large number and favoring crossing of the BBB	[99,100]	The age of tissue/cell donors influences the number of ASCs, and thereby EVs, obtainable from SAT, even though the results are still conflicting	[44,75,101]
Low/null expression of membrane histocompatibility markers, reducing the risk of host immune responses	[99]	Weight of donors and the presence of metabolic pathologies can alter EVs’ quality/characteristics	[29,101,102]
EVs carry only a fraction of the molecules produced by the cells of origin, favoring selection for specific therapeutic purposes	[100]	Restriction in the range of products donated by EVs compared with entire MSCs, so that the therapeutic dose and efficacy of EVs must still be clearly defined	[92]
Homing certain tissues depends on their source tissue, which would be mainly useful in the case of the use of EV for targeted drug delivery	[33]	It is not clear how extensive the EV homing ability is.	[33]
EVs, similar to iPSCs and unlike MSCs, should not be tumorigenic and, in selected cases, could also serve as a new anticancer tool	[103,104,105]	Data are still conflicting. One should not forget the angiogenic properties of EVs, which may favor tumor growth/expansion or the development/worsening of metabolic diseases	[51,105,106,107]

Abbreviations: ASCs, adipose stromal/stem cells; BBB: blood–brain barrier; EVs, extracellular vesicles; iPSCs, induced pluripotent stem cells; MSCs, mesenchymal stem cells, SAT, subcutaneous adipose tissue.

## Data Availability

Not applicable.

## Abbreviations

ADicerKO	knockout of the miRNA-processing enzyme Dicer
ALS	amyotrophic lateral sclerosis
AMPK	AMP-activated protein kinase
ASCs	adipose stromal/stem cells
AT	adipose tissue
BAT	brown adipose tissue
BBB	blood–brain barrier
BM-MSCs	bone marrow-derived MSCs
CT	clinical trial
DIO	diet-induced obesity
Dlk1	Notch1 inhibitor delta-like 1 homolog
EVs	extracellular vesicles
FAs	fatty acids
FABP4	fatty acid binding protein 4
FGF2 or 21	fibroblast growth factor 2 or 21
FIH-1	inhibitor of hypoxia-inducible factor 1
GMPs	good manufacturing practices
HIF-1(α)	hypoxia-inducible factor 1(α)
IBD	inflammatory bowel disease
IGF-1	Insulin like growth factor 1
iPSC	induced pluripotent stem cells
MAPK	mitogen-activated protein kinase
miRNA(s)	microRNA(s)
MMP-2	matrix metalloproteinase 2
MSCs	mesenchymal stromal/stem cells
mTOR	mammalian target of rapamycin
N/A	not available
NF-kB	nuclear factor kB
PDGF	platelet-activated growth factor
PI3K	phosphatidylinositol 3-kinase
PPARγ	peroxisome proliferator-activated receptor γ
PPIK	PI3K-related protein kinases
TGF-ß	transforming growth factor ß
mTORC1	mTOR complex 1
mTORC2	mTOR complex 2
SAT	subcutaneous adipose tissue
VAT	visceral adipose tissue
VEGF	vascular endothelial growth factor
WAT	white adipose tissue
